# The emergent rhizosphere: imaging the development of the porous architecture at the root-soil interface

**DOI:** 10.1038/s41598-017-14904-w

**Published:** 2017-11-01

**Authors:** J. R. Helliwell, C. J. Sturrock, S. Mairhofer, J. Craigon, R. W. Ashton, A. J. Miller, W. R. Whalley, S. J. Mooney

**Affiliations:** 1 Division of Agricultural and Environmental Sciences, Gateway Building, Sutton Bonington Campus, University of Nottingham, Leicestershire, LE12 5RD UK; 2 Metabolic Biology, John Innes Centre, Norwich Research Park, Norwich, NR4 7UH UK; 3 Sustainable Soils and Grassland Systems Department, Rothamsted Research, West Common, Harpenden, Hertfordshire, AL5 2JQ UK

## Abstract

The rhizosphere is the zone of soil influenced by a plant root and is critical for plant health and nutrient acquisition. All below ground resources must pass through this dynamic zone prior to their capture by plant roots. However, researching the undisturbed rhizosphere has proved very challenging. Here we compare the temporal changes to the intact rhizosphere pore structure during the emergence of a developing root system in different soils. High resolution X-ray Computed Tomography (CT) was used to quantify the impact of root development on soil structural change, at scales relevant to individual micro-pores and aggregates (µm). A comparison of micro-scale structural evolution in homogenously packed soils highlighted the impacts of a penetrating root system in changing the surrounding porous architecture and morphology. Results indicate the structural zone of influence of a root can be more localised than previously reported (µm scale rather than mm scale). With time, growing roots significantly alter the soil physical environment in their immediate vicinity through reducing root-soil contact and crucially increasing porosity at the root-soil interface and not the converse as has often been postulated. This ‘rhizosphere pore structure’ and its impact on associated dynamics are discussed.

## Introduction

The physical characteristics of soil surrounding roots regulate many key plant processes and interactions including water uptake, nutrient acquisition, gaseous exchange and microbial proliferation. Soil immediately adjacent to the root has very different physical, chemical and biological properties when compared with the bulk soil^1,2^. The zone of soil around the root that is altered by plant activity is defined as the rhizosphere. Many studies have reported distinct and unique differences with soil from the root-soil interface, including soil structure^3,4^, hydraulic properties^5^ and mechanical stability^6^. Likewise the role of exudates and associated microorganisms in affecting rhizosphere biochemistry^1,2^ and biophysics^7–9^ is well reported and widely considered beneficial to root proliferation.

Previous empirical models and experimental work have predicted root growth can cause densification in the rhizosphere due to radial expansion^10^ and significantly reduce soil porosity. Aravena *et al*.^5,11^ showed root induced soil compaction can increase root-soil contact which has key implications for hydrological behaviour which they demonstrated by modelling approaches. Whether the physical structure of the rhizosphere is changed by natural processes such as particle rearrangement during root expansion^3^; shrinkage during water extraction^12–14^; mucilage exudation influencing soil water affinity^15–17^; or anthropogenic impacts such as agricultural seedbed preparation^18^ and vehicular traffic^19^, there are generally important implications for root-soil processes.

To date relatively few direct investigations, such as Schmidt *et al*.^20^, have attempted to study the undisturbed rhizosphere as an entire entity i.e. *in-situ* without attempting to disentangle isolated processes. Thin-section microscopy techniques have enabled the exploration of rhizosphere physical^21^ and hydraulic properties^22^. North and Nobel^23^ successfully used field impregnated thin sections to measure root-soil contact in *Opuntia ficus-indica* under drought conditions to assess the impact of the rhizosheath on hydraulic conductivity. More recent advances in nano-scale secondary ion mass spectrometry have enabled the extensive exploration and imaging of nitrogen use in the rhizosphere^24,25^. However both techniques are to an extent laborious, require substantial root and soil disturbance, and do not allow for the dynamic study of living roots over time. Non-destructive imaging such as X-ray radiography^26^, X-ray Computed Tomography (CT)^11,27,28^, and Neutron Radiography^17^ are enabling us to overcome these limitations by providing undisturbed, rapid visualisations of the soil and root environment at a wide range of rhizosphere relevant spatial resolutions (µm to mm).

This study focused on the biophysical impact of root growth on the micro-scale structural development in aggregating soil and creating pore space during the early stages of rhizosphere formation. Specifically, we aimed to: i) image the pore structure of the rhizosphere *in-situ* in unstructured (e.g. sieved) soil collected from the field; ii) assess how early-stage root development influences the physical architecture of the rhizosphere over multiple time-points; and iii) investigate the effect of soil texture i.e. soil particle size on rhizosphere development and assess specifically how the pore morphology around a growing root system relates to known functions of different parts of root axis. We used X-ray micro CT to image the development of soil micro-structure surrounding living roots in three dimensions (3-D), offering new insights into the speed and scale at which the architectural arrangement of the rhizosphere takes shape.

## Materials and Methods

### Experimental Design

Four replicate columns (70 mm height × 25 mm diameter), per soil texture, were uniformly packed to a bulk density of 1.2 Mg m^−3^ with air dried soil sieved to ≤2 mm. The soils used were a Newport series loamy sand (sand 83.2%, silt 4.7%, and clay 12.1%; pH 6.35; organic matter 2.93%; FAO Classification: typical brown sand) and Worcester series clay loam (sand 35.6%, silt 31.5%, and clay 32.9%; pH 6.50; organic matter 5.19%; FAO class: Argillic Pelosol) soil from the University of Nottingham farm at Bunny, Nottinghamshire, UK (52.52°N, 1.07°W). The water retention curves and selected physical properties for these soils are provided as supplementary information (Supplementary Figures 1 and 2). The samples were adjusted to and maintained at required matric potentials with tension table apparatus modified from Chapman *et al*.^29^. Microcosms were initially saturated with deionised water before being placed at −2 kPa until equilibrated (c. one day), and then the matric potential lowered to −8 kPa for three days. Following this microcosms were returned to −5 kPa (over c. two days) and maintained at this tension through seedling establishment and growth. This process of wetting-drying-rewetting took approximately one week and was important to stabilise the soil structure and minimise slumping so we could isolate changes associated with root growth, without inducing any noticeable cracking due to soil shrinkage. Our pilot investigations showed one wetting and drying cycle as described above was optimal for stabilising soil structure and further wet-dry cycles had no significant effect on soil porosity measurements. Surgical micropore tape (3 M United Kingdom PLC, Bracknell) was placed over the columns during soil preparation to reduce soil surface evaporation and prevent sample contamination from the atmosphere, whilst still enabling gaseous exchange. Seeds of tomato (*Solanum lycopersicum*) *cv*. ‘Ailsa Craig’ were germinated on moist filter paper for 48 hours before being planted 5 mm below the soil surface. Plants were grown in a controlled environment room (27 °C day/22 °C night); 40% relative humidity; a 12 hour photoperiod with a photosynthetic photon flux density at plant level of 330 µmol m^−2^ s^−1^ in a climate chamber. The mass of each sample was recorded daily to ensure the moisture content was maintained.

### X-ray CT scanning

All samples were scanned using a Phoenix Nanotom 180NF X-ray micro-CT scanner (GE Sensing and Inspection Technologies, Wunstorf, Germany). The source had a 3 µm focal spot, with the centre of the sample 5.4 cm from the X-ray source and a resultant imaged voxel size of 12 µm. The entire sample was imaged with of field of view 2308 × 2308 pixels, X-ray energy of 115 kV, current of 110 µA and an exposure time of 750 ms. A 0.2 cm Cu filter was used and 1600 image projections (appropriate for this system) were taken, at an averaging of 3 and skip of 2 images per projection. Each scan took 70 minutes to complete. Although faster scan times are possible we chose this duration to optimise the image quality. Each sample was scanned at zero, two, four, six and eight days after planting (equivalent to 40 scans in total), exposing each plant to a calculated dose of 7.55 Gy per scanning session^30^ and equating to a total dose of 37.8 Gy over the eight day incubation. All images were cropped 3 mm from the column edge after tomographic reconstruction to remove soil near the container sides to minimise potential edge effects (note, roots were very rarely observed in this area) and subsequently processed in VG StudioMax^®^ 2.1 software.

### Statistical analysis

All data were analysed in GenStat Release 15.1 (VSN International) using repeated measures single-variate mixed models (REML), to isolate the effects of sampling period on X-ray CT measures of the rhizosphere structure over the eight day incubation, with a combination of sample and distance from root surface as a random effect. Standard residual plots were examined in GenStat to check data normality.

### Data availability

The datasets generated and analysed during this study are available from the corresponding author on request.

## Results

### Development of the Imaging Approach

**Figure 1:**
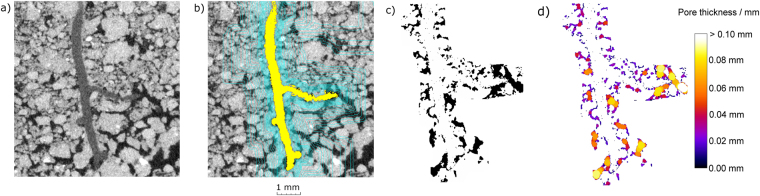
An overview of the experimental protocol used to isolate regions near and away from the root surface: (**a**) a raw greyscale image of a root growing through soil; (**b**) root material segmented in yellow, with multiple voxel dilations highlighted in blue; (**c**) the segmented pore space surrounding the root shown in black, for a single dilated region; (**d**) heat map of pore thickness, giving an indication of 3-D pore diameter.

The aim of the imaging approach was to isolate three key components in each X-ray CT derived image namely soil matrix (including soil minerals and fine organic material), root material and air and water filled pore space (hereafter referred to as pore space). Qualitative visual inspection of the CT images showed clear feature boundaries which aided the segmentation of phases and regions of interest (Fig. 1 and Supplementary Figure 3). Segmentation of tomographs was performed after applying a median filter of 3 pixel radius to the data (Fig. 1a), to reduce point noise, but preserve structural borders^31^. To assist with separating solid material from surrounding pore space, the greyscale histogram was calibrated against the soil pore space and a common aluminium reference object present in all scans using the surface calibration algorithm within VGStudioMAX (version 2.1) software (Volume Graphics GmbH, Heidelberg, Germany). This tool uses a global thesholding approach by applying a surface at the grayscale value equal to 50% of the range between background (air space) and a material (aluminium reference object) sampled regions. The aluminium reference object was 5 mm × 3 mm × 0.5 mm and chosen as it had a similar grayscale value to the soil matrix. Its use enabled a common threshold value to be applied relative to specific X-ray attenuation across all scans at all time points. A region of interest was subsequently generated from this surface and defined as soil matrix. This soil matrix region was then inverted and the segmented root material subtracted, in order to isolate soil pore space and root material. Roots were segmented from the soil matrix using an adaptive region growing algorithm in VGStudioMax software, which starts from a fixed point containing known properties (i.e. the greyscale value of the desired feature) and includes all common surrounding voxels within a specified selection. Whilst the observed image quality seemed appropriate for the study, and image blur, an important source of error^32^, was minimal, we chose as a precaution to exclude all data within one voxel of the root surface to compensate for any partial volume effects or mis-segmentation of the root system itself (Supplementary Figure 3).

**Figure 2:**
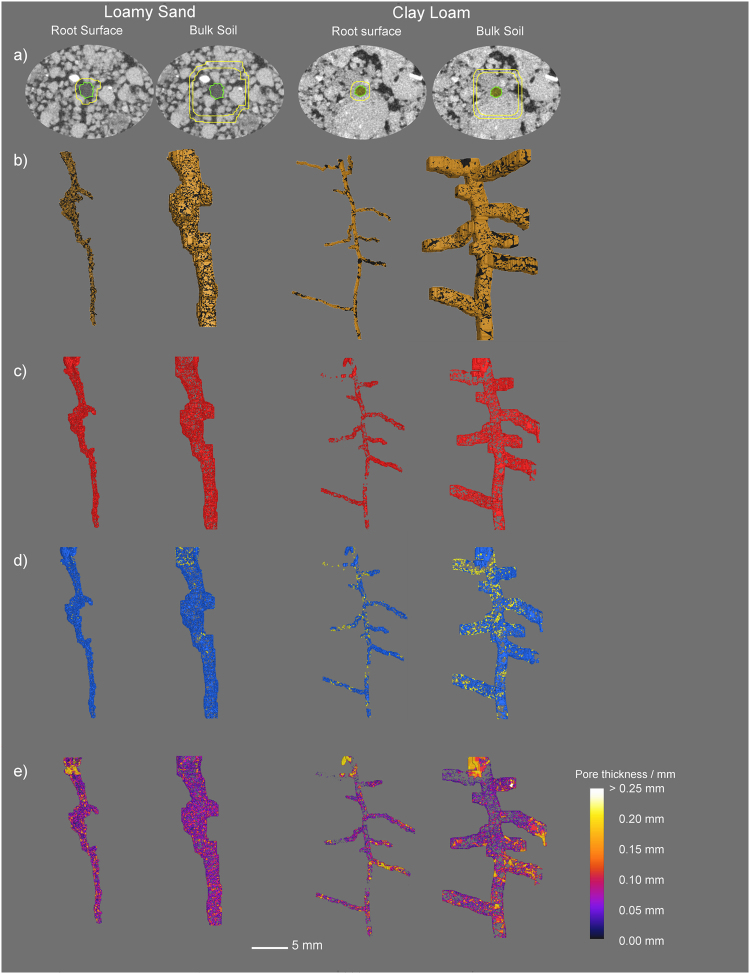
Demonstration of the image analysis protocol showing segmentation of an isolated region of the root-soil complex at the root surface (between 0.024 and 0.12 mm from the root surface) and from the bulk soil (between 0.48 and 0.6 mm from the root surface) for sand and clay soil textures: (**a**) filtered greyscale image with segmented root material shown in yellow, and isolated region of interest highlighted; (**b**) segmentation of the solid soil material (brown) and pore (black) material at eight days of growth; (**c**) the fully isolated pore system shown in red; (**d**) the pore system classified separately as macropores (blue) and mesopores (yellow); (**e**) the pore system classified based on the thickness of the soil pores.

In order to assess soil structural changes with increasing distance away from the root surface, a new imaging protocol was developed. The segmented root region was dilated in 3-D with increasing two voxel (24 µm side length) iterations to a total of twenty voxels (240 µm) dilations, and then every five voxels (60 µm) up to fifty voxels (600 µm) (Fig. 1b). A further dilation of 1 mm from the root surface was made to ensure the spatial scale was appropriate. This resulted in seventeen discreet regions moving away from the root surface for each scan, into which the corresponding soil, root and pore space segmentations could be inserted (Fig. 1c; Fig. 2b,c). A further dilation of one voxel from the root surface was undertaken and the soil and pore segmentations subtracted from the first two voxel dilations, in order to account for any potential mis-segmentation of the root section associated with partial volume effects^32^. The volume of pore and soil material within each of the dilated regions was calculated from which the 3-D porosity of each region moving away from the root could be ascertained.

In order to assess how the porosity of each of the seventeen dilated regions altered with increasing depth down the root axis, images were false-coloured so that the pore space of the dilated region was ‘black’, with all other image features (solid material within the dilated region, root material, pore and solid material outside of the dilated region) shown as ‘white’. A top-down (XY) image stack of 1050 images for each scan was exported, with each image corresponding to a 24 µm increase in depth. This process was repeated, but with solid material within each dilated region false coloured as ‘black’ and all other features ‘white’. Coupled with the pore image stack, this gave an independent map of the pore and solid material within each dilated region. A total of 34 image stacks were exported per sample per day of growth (one pore and one soil stack per dilated region), generating 142,800 images for analysis. Each binarized image stack was approximately 4 GB in size. The total and individual area of all pore and solid material for each image was calculated, from which the porosity along the root axis at 24 µm intervals was also calculated using the particle analyser function in ImageJ.

Temporal changes to the rhizosphere pore structure were measured by analysing pore space within each region moving away from the root surface via two approaches: 1) pore characteristics within discreet regions were analysed individually (i.e. 24–48 µm, 48–72 µm, 72–96 µm etc.) and 2) pore characteristics within compounded (i.e. cumulative) regions moving away from the root surface (i.e. 24–48 µm, 24–72 µm, 24–96 µm etc.) were analysed. Data were also presented as two discreet distances from the root surface, defined as 24–120 µm (‘root surface’) and 480–600 µm (‘bulk soil’) (Fig. 2). At each time point, 3-D pore thickness was assessed^33^ (Figs 1 d, 2e). Individual macropores (diameter > 72 µm) and mesopores (diameter 24–72 µm) within each sample were isolated based on their corresponding pore thickness (Fig. 2d), allowing the evaluations of changes to overall number and volume of defined pore classes over time. To confirm the validity of the approach and ensure the soil structural dynamics could be directly attributed to root growth, a separate control experiment using an artificial root (in this case nylon wire) was performed on both soil types and analysed using the method described above (Supplementary Figures 4 and 5).

### The impact of root growth on soil structural dynamics

**Figure 3:**
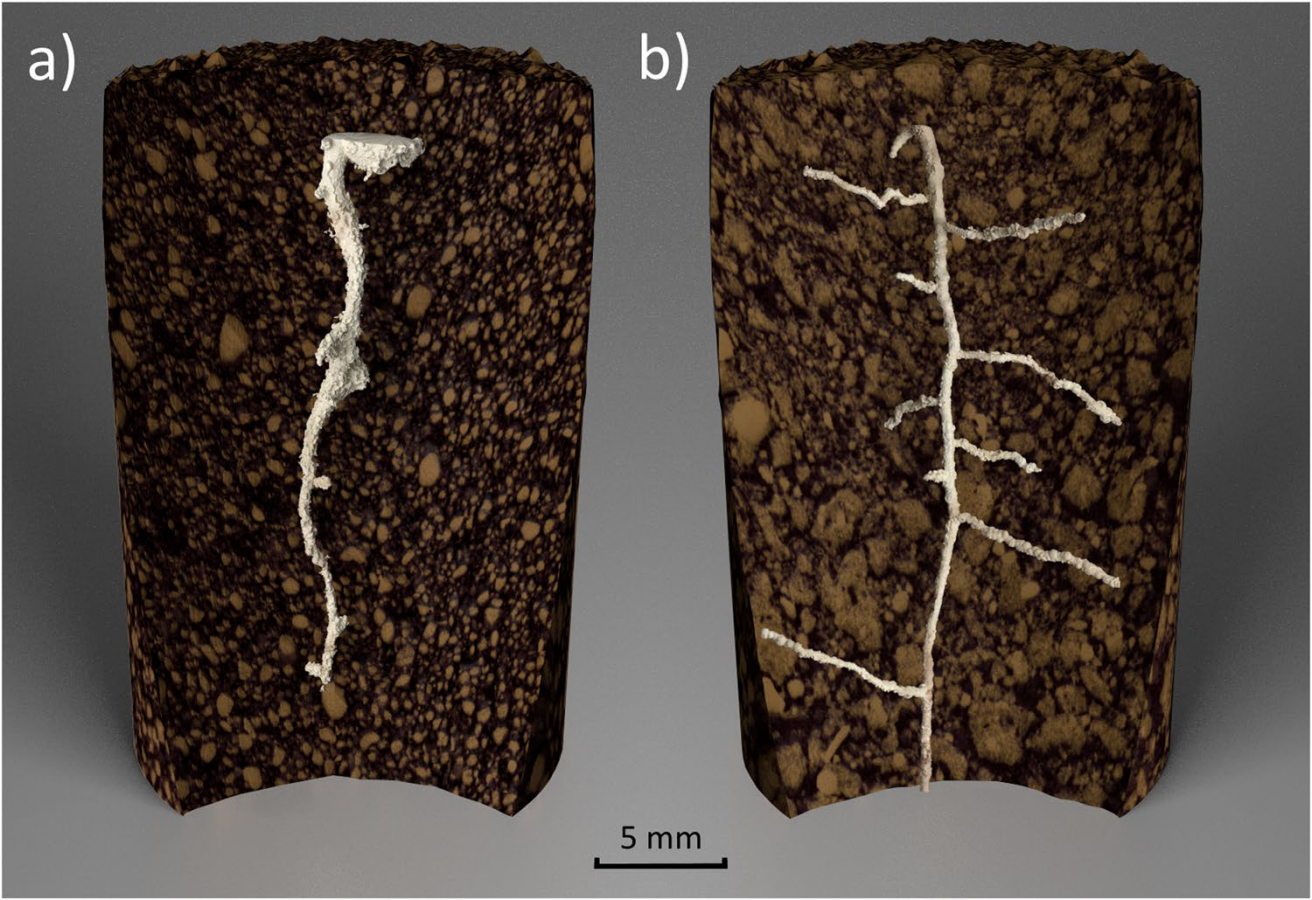
Examples of full segmented root systems (pale yellow) as embedded in the soil (brown) and pore space (black) at eight days of growth, for: (**a**) sand; and (**b**) clay soil textures.

Porosity was measured in all scans from day 0 and revealed homogeneity in the packing procedure allowing us to confirm future changes in soil pore structure could be attributed to the emerging root system (Supplementary Figure 5a). Soil texture was shown to influence developing root architecture. Plants grown in the loamy sand texture exhibited significantly thicker roots (P < 0.001, mean thickness of 0.708 ± 0.047 and 0.415 ± 0.018 mm diameter, respectively after eight days growth) with a higher total root volume than the clay loam soil (mean volumes of 11.54 ± 2.03 and 6.98 ± 0.59 mm^3^ respectively after eight days of growth). Furthermore, roots grown in the clay loam soil exhibited higher numbers of lateral roots (Fig. 3), which was confirmed qualitatively by destructive sampling and root washing at the end of the experiment.

**Figure 4:**
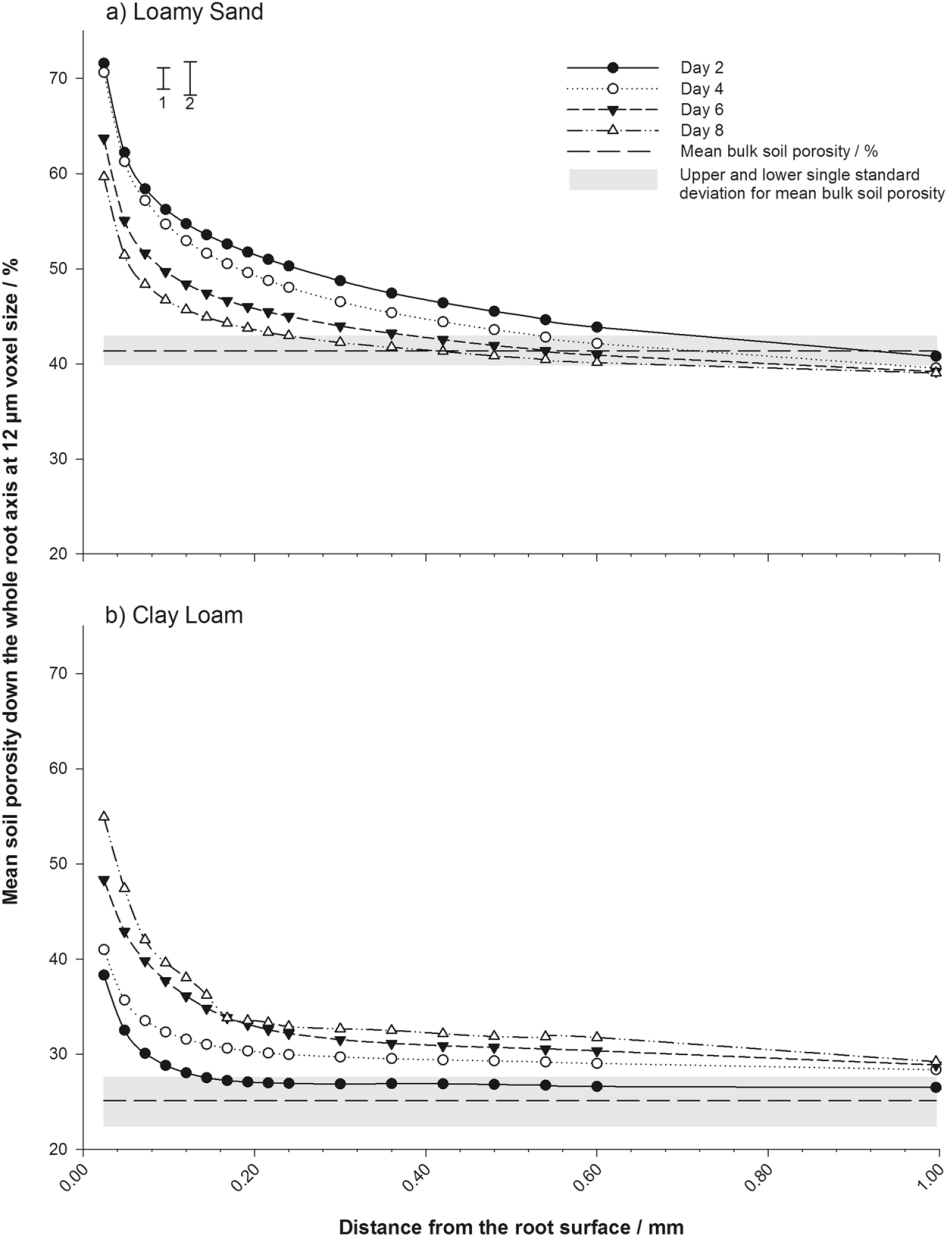
Variations in soil porosity over time for: (**a**) sand; (**b**) clay soils moving away from the root surface. Values represent the mean of four replicates. Standard errors of the difference are shown for: (1) sampling day; (2) soil texture.

There was a clear gradient in the rhizosphere pore structure immediately adjacent to the tomato roots after two days of growth, with porosity over all days significantly higher at the root surface compared to the bulk soil in both clay loam and loamy sand (P < 0.05) soils. However the degree of structural change at the root surface was governed by soil texture and time (Fig. 4). The loamy sand exhibited a much greater increase in rhizosphere porosity compared to the bulk soil than the clay loam. At day two, porosity immediately at the root surface in the loamy sand texture was 71.6% at the root surface compared to 41.3% in the bulk soil and 38.3% at the root surface in the clay loam compared to 25.1% in the bulk soil. With time the sand soil consolidated, and by day eight the degree of porosity difference between the soil at the root interface compared to the bulk soil reduced to 18.7%. Conversely over time the rhizosphere pore structure in the clay loam became less dense, with porosity adjacent to the root 29.8% higher than in the bulk soil by day eight. In contrast, Supplementary Figure 5b shows for an artificial root system there was only a very modest increase (c. 3%) in porosity at the interface between soil and ‘root’ followed by a small localised (c. 0.06 mm from the root) compaction characterised by a reduction in soil porosity (T0 = 24.7% and T1 = 22.1%).

There was a strong relationship with the rhizosphere pore structure measured along the whole root axis and with distance from the root surface, which was soil texture dependent. There was a significant effect of distance from the root (P < 0.001) and soil texture (P < 0.001) on soil porosity as an individual factor, and a significant interaction between time and texture (P < 0.001). The loamy sand soil exhibited a larger root influenced structural zone (0.72 mm) after day two, decreasing in size with time (0.24 mm) from the root surface by day eight (Fig. 4a). This decrease in size also complemented a change in porosity gradient. At day two the gradient in porosity was at its shallowest, increasing with time and forming the strongest gradient by day eight. Conversely in the clay soil (Fig. 4b), the size of the root-influenced structural zone increased from 0.16 mm at day two to >1 mm by day eight. Similarly a changing spatial size paralleled a changing structural gradient moving from the root surface over time. Day two exhibited the steepest gradient in soil porosity, declining in magnitude over time to a shallower gradient spanning over 1 mm from the root surface by day eight.

### Impact of a maturing root system on the emerging rhizosphere structure

**Figure 5:**
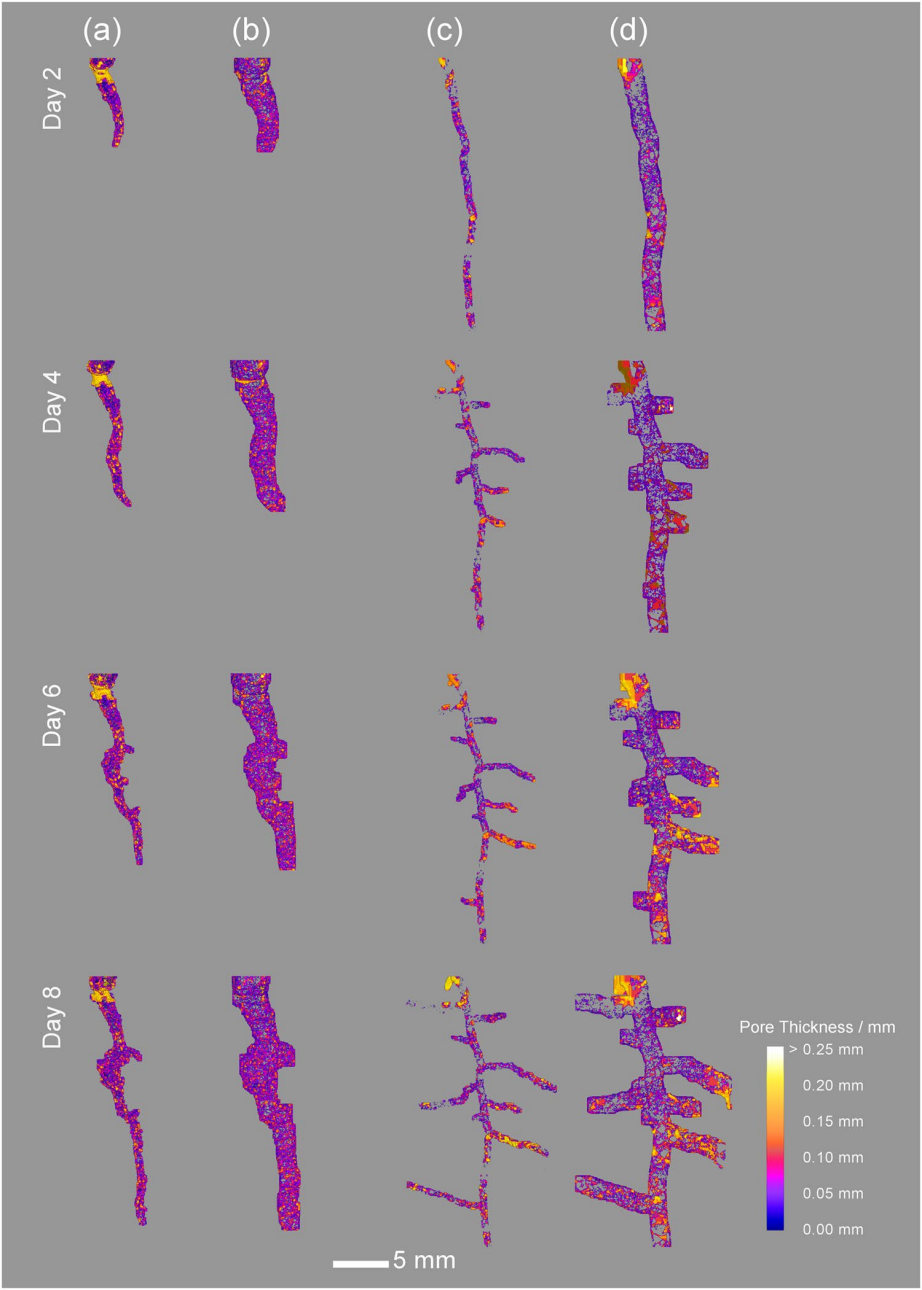
Thickness distributions of soil pores in the sand (**a,b**) and clay soil (**c,d**) at the root surface (**a,c**) (between 0.024 and 0.12 mm from the root surface) and bulk soil (**b,d**) (between 0.48 and 0.6 mm from the root surface) with time.

Measurement of soil pore thickness offers a unique way to characterise changes to the 3-D rhizosphere pore structure through space and time and across treatments, by accounting for the entire sample. Changes to pore thickness relate to the zones of influence within the rhizosphere observed in the overall mean porosity plots, with large increases in pore thickness in both the clay loam and loamy sand (Fig. 5) confined to the first 300 µm from the root surface suggesting this is a crucial zone.

At the root surface there was a significant temporal change in number of macro- and mesopores (P < 0.05) and 3-D pore volume (P < 0.05) between day two and day eight in the clay soil (Fig. 2d), and demonstrated in Fig. 5. At the root surface in the clay loam soil, there was an increase in mean number of total macropores over time, from 8 (±1.4) to 55 (±8.7) between days two and eight respectively. Likewise macropore number/mm^3^ of soil increased from 1.01 (±0.11) to 2.27 (±0.18) under the same conditions. This corresponded to a 700% increase in total macropore volume/mm^3^ clay loam soil over the eight day incubation, increasing from 5 × 10^−3^ (±2 × 10^−3^) to 4 × 10^−2^ (±9 × 10^−3^) mm^3^/mm^3^. Likewise mesopore characteristics changed over the incubation period in the clay loam soil. Mean total number of mesopores increased from 34 (±2.7) to 349 (±72.3) between days two and eight respectively at the clay soil root surface, whilst the number of mesopores/mm^3^ increased from 4.47 (±0.52) to 13.99 (±0.83) over the same time period. This corresponded to a 225% increase in total mesopore volume/mm^3^ between days two and eight, changing from 4 × 10^−4^ (±6.3 × 10^−5^) to 1.3 × 10^−3^ (±5.6 × 10^−5^) mm^3^/mm^3^. There was no significant change in the total number of macropores or macropores/mm^3^ in the sandy loam soil at the immediate root surface over time, but mean macropore volume/mm^3^ increased by 145% from 1.1 × 10^−2^ (±2.2 × 10^−3^) to 2.7 × 10^−2^ (±4.3 × 10^−3^) mm^3^/mm^3^ between days two and eight. Furthermore the total number of mesopores at the sandy loam soil root surface increased from 3 (±1.3) to 23 (±9.6), and corresponded to an increase in mesopore number/mm^3^ from 0.30 (±0.12) to 1.17 (±0.34) over the incubation. Mean mesopore volume/mm^3^ in the sandy loam soil increased by 276% from 2.5 × 10^−5^ (±1.3 × 10^−5^) to 6.9 × 10^−5^ (±1.9 × 10^−5^) mm^3^/mm^3^ between days two and eight respectively (Fig. 5).

**Figure 6:**
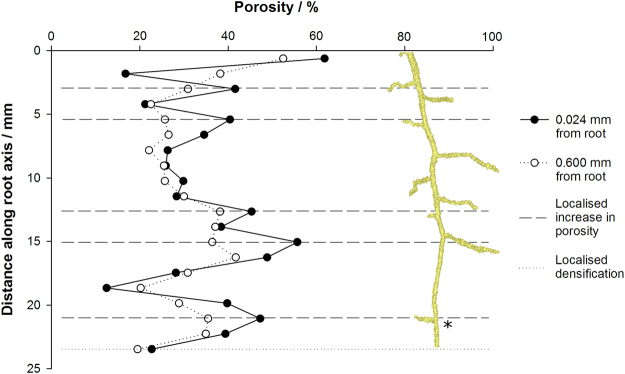
Root induced changes to soil porosity with depth down the root axis, mapped at the immediate root surface (0.024 mm from the root) and in the bulk soil (0.6 mm from the root) eight days after emergence highlighting zones of increased porosity and densification specific to root classification. The zone of root elongation is indicated by an asterisk symbol.

Crucially we have shown the physical architecture of the rhizosphere soil is different at selected positions along a root axis, which has not previously been measured non-invasively. We noted variations in pore thickness at discreet zones along the root axis (Fig. 5). In particular, the tip of lateral roots and the area surrounding the zone of elongation, exhibited much larger increases in pore thickness compared to the other positions along the root. The spatial size to which these trends hold appear to be governed by soil texture (i.e. a function of particle size), with the clay loam soil exhibiting a greater variation in pore thickness than the sandy loam soil across all time points (Fig. 5). Whilst within treatment variation was high with depth due to variability in lateral root proliferation, our results show the zone immediately at the root surface exhibited higher porosity than bulk soil measurements, regardless of position along the root axis (Fig. 6). The rhizosphere pore structure surrounding the zone of elongation exhibited an increase in porosity of 11.8% compared to bulk soil at a distance of 2.4 mm from the root tip (Fig. 6). Likewise we observed localised increases in porosity surrounding lateral roots (dashed horizontal lines in Fig. 6), although not all lateral roots responded in the same way. Densification of the rhizosphere soil was clearly apparent around the primary root tip, extending to 1 mm up the root axis (dotted horizontal line in Fig. 6).

## Discussion

In both soils the porosity adjacent to the root was significantly higher than in the bulk soil (Fig. 4). Within 1 mm from the root the porosity reduced exponentially to the value of the bulk soil. This effect of roots on the surrounding soil is different from previous work such as^34^, where a reduction in porosity was found immediately adjacent to the root using radiographic methods. They argued that the decrease in porosity was due to soil compression as the root expanded. One possible reason as to why we observed this zone of increased porosity at the root interface is the high resolution we employed (12 µm) compared to other studies such as 20. The increase in porosity adjacent to the root we found is consistent with localised particle/micro-aggregate rearrangement following the combined influence of shear and compressive forces generated by the mechanisms of root growth^35^. Supplementary Figure 4 illustrates the structural generation at the root interface is quite different when comparing between a natural and artificial system. In addition we note that Braunack and Freebairn^34^ equilibrated the soil to −10 kPa compared to −5kPa in this work; hence it is possible differences in matric potential which affect shear and compressive strength may account for contrasting observations.

X-ray CT permits us to track the temporal changes in porosity. In the sandy soil, following the initial increase in porosity (between the day zero and two), porosity decreased between days two and eight. This is consistent with the idea of soil compression by an expanding root^34,36^. Although the initial (up to day two) increase in porosity in the clay loam soil was much smaller than in the sandy loam (Fig. 4). The porosity of the clay loam soil continued to increase until day eight, which we attribute to the effect of water extraction by the root. Compared to the clay loam, the sandy loam soil had only a limited ability to shrink (Supplementary Figure 2). In the clay loam soil water uptake is likely to result in localised spatial variation in water content and these effects can lead to the formation of micro-cracks, which would explain the continued reduction in rhizosphere porosity up to day eight (Fig. 4). The radial extent of the rhizosphere in the clay loam soil was greater than that in the sandy loam soil. This is also consistent with our explanation for the different mechanisms responsible for developing the rhizosphere pore structure in the two soils between days two and eight.

We observed that the pore structural changes in the rhizosphere were not uniformly distributed around the root (Figs 2, 4, 6), with distinct zones along the root axis impacting on the rhizosphere pore structure in different ways. Valentine *et al*.^37^ found lateral roots in a clay soil exhibited a clear root growth strategy proliferating through existing pore channels in search of water and nutrients. We also observed early root proliferation associated with pore structure in the soil, which were subsequently beneficially utilised as sites for lateral root emergence. Agar plate and rhizotron studies have reported lateral root initiation depends on localised nutrient supply^29,38,39^. However the proliferation of lateral roots into soil pores suggests a potential thigmotropic response, which may be missed in agar, hydroponic and possibly even sand culture experiments. The temporal changes to pore thickness mapped in Fig. 5 support the hypothesis that lateral roots can be initiated at sites of decreased porosity, with increases in pore thickness observed immediately at the root surface around lateral roots compared to other parts of the root axis further suggesting proliferation into pre-existing pores. Pore thickness in these regions subsequently increased with time, indicating that physical processes surrounding the lateral root regions in isolation account for this temporal change. This idea is supported by Fig. 6, where increases in porosity at depth correspond with regions along the root axis in which lateral roots are present. These results indicate how particle size and subsequent moisture content can have a profound effect on the development of root systems architecture through evolution of the surrounding rhizosphere pore structure. It has been suggested that lateral roots as opposed to tap roots are primarily responsible for water uptake in plants^40,41^. Hence, we hypothesise that we are observing the effects of localised wetting and drying cycles in the rhizosphere even though soil moisture content was maintained at a constant value throughout our experiment. Under moist conditions such as those in this investigation, it is widely acknowledged the clay constituents in soil swell^42^, increasing inter-aggregate contacts and creating increased hydraulic conductivity among aggregates^43^. Hence, this decrease in contact surrounding the root would be expected to have important implications for water and solute movement, by reducing the water capillary potential of the surrounding aggregates, but maintaining the pore continuity within the rhizosphere and improving oxygen and carbon dioxide movement to and from the root system respectively. Both factors are important for plant growth, and require further study if modelling approaches are to be correctly utilised.

Constraints of the present imaging approach include the comparatively small column size, limiting the investigation of young plants as the root systems beyond this become pot bound, and the significant computing hardware limitations in handling such large datasets (raw data *c*. 25 GB per sample). Furthermore the imaging resolution (12–24 µm) prevented the quantification of root hairs (c. 15–18 µm diameter), which are known to extend beyond the rhizosphere pore structure reported here and impact on aggregate stability^44^ and nutrient acquisition^45^.

## Conclusions

The rhizosphere rapidly develops its own structure with a significant gradient in the soil porous architecture during early root growth compared to the bulk soil, which we have microscopically mapped and, crucially, explored dynamically for the first time in 3-D by imagery. The nature of this rhizosphere pore structure varied depending on soil texture, but generally develops quickly around a growing root (i.e. within two days) creating a highly porous region at the root:soil interface and further matures over time. In addition, we found the physical architecture of the porosity within the rhizosphere has a number of structurally distinct zones some of which are far smaller than known biological and chemical gradients surrounding growing plant roots, i.e. within the order of μm as opposed to mm. We have shown that initially the porosity of the rhizosphere soil adjacent to the roots increased in contrasting soils but thereafter, it decreased in a sandy loam soil whereas it increased in a clay loam soil. The temporal changes in this rhizosphere pore structure were consistent with expected differences considering in the conventional physical behaviour associated with these soils. These results highlight the importance of the representing the microscopic soil physical environment, and in particular, the different soil structural regions that exist within the rhizosphere in future modelling efforts concerning the behavioural dynamics of the rhizosphere particularly those concerned with water, nutrient and gas flows around plant roots.

## Electronic supplementary material

Supplementary Figures
