# Supplementary material for: The emergent rhizosphere: imaging the development of the porous architecture at the root-soil interface
Source: Sci Rep. 2017 Nov 1;7:14875. doi: 10.1038/s41598-017-14904-w (PMC5665926; doi:10.1038/s41598-017-14904-w)

The emergent rhizosphere: imaging the development of the porous architecture at the root-soil interface

J. R. Helliwell[1,3], C. J. Sturrock[1], S. Mairhofer[1], J. Craigon[1], R. W. Ashton[3], A. J. Miller[2], W. R. Whalley[3] & S. J. Mooney*[1]

[1]*Division of Agricultural and Environmental Sciences, Gateway Building, Sutton Bonington Campus, University of Nottingham, Leicestershire, LE12 5RD, U.K.*

[2]*Metabolic Biology, John Innes Centre, Norwich Research Park, Norwich, NR4 7UH, UK*

[3]*Sustainable Soils and Grassland Systems Department, Rothamsted Research, West Common, Harpenden, Hertfordshire, AL5 2JQ, U.K.*

*Corresponding author: Sacha J. Mooney
Division of Agricultural and Environmental Sciences, Gateway Building, Sutton Bonington Campus,
University of Nottingham, Leicestershire, LE12 5RD, UK

E-mail: sacha.mooney@nottingham.ac.uk

Supplementary Figures

Figure S1 – Water release characteristics of the loamy sand and clay loam soil textures used in this investigation. The water release characteristic was measured using pressure chamber apparatus, and fitted to the van Genuchten-Mualem model (1980). Error bars represent standard error of 3 replicates.

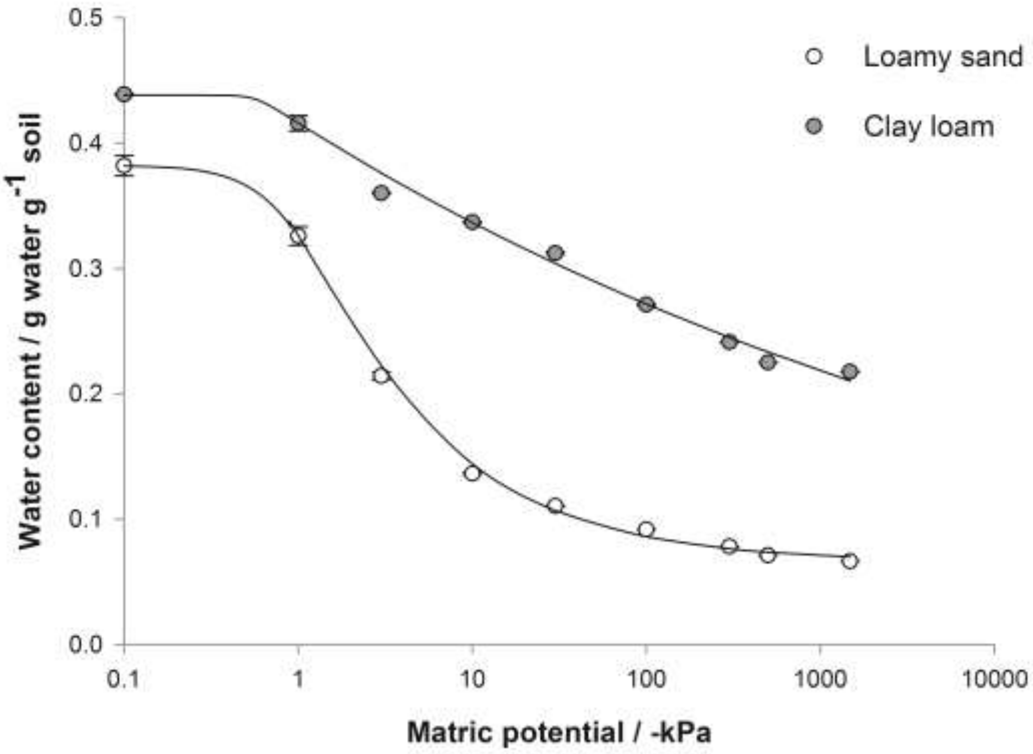

Reference

van Genuchten MT. (1980). A Closed-form Equation for Predicting the Hydraulic Conductivity of Unsaturated Soils. Soil Sci Soc Am J **44**, 892-898.

Figure S2 – Penetrometer resistance and linear shrinkage of the loamy sand and clay loam soil textures used in this investigation. Error bars represent standard error of 3 replicates.

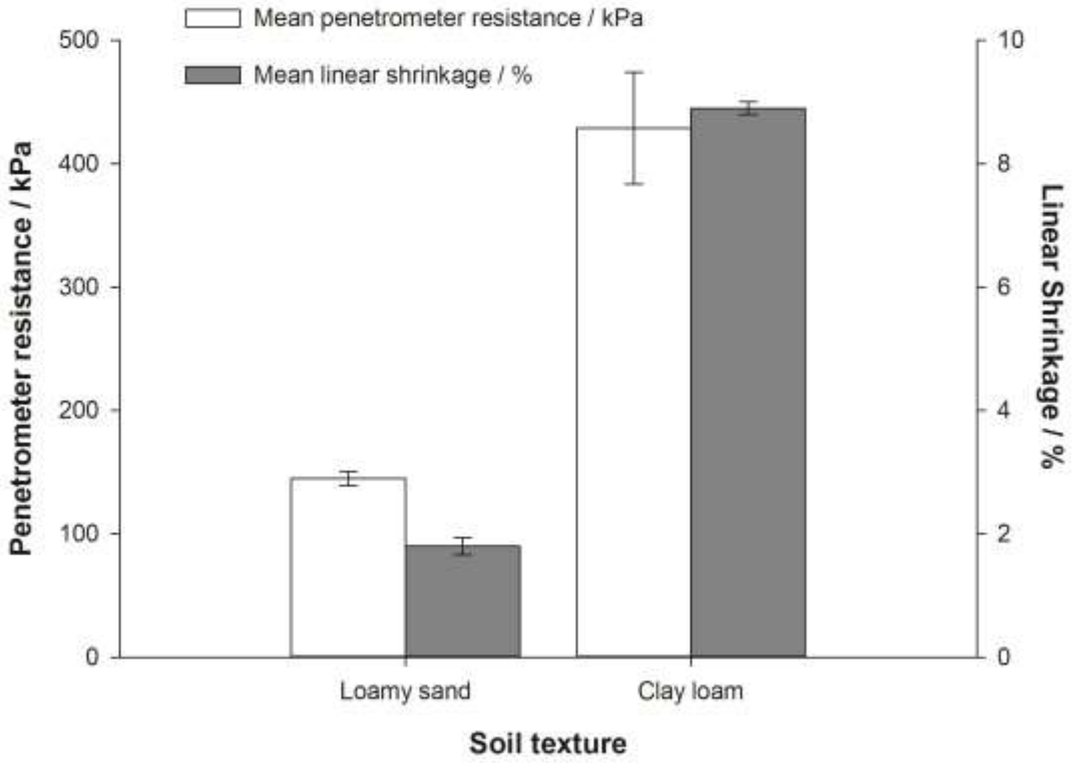

Figure S3 – Schematic diagram showing the full image analysis procedure. (A) Unprocessed CT image in XY plane; (B) Median (3x3) filtered CT image (C); Image showing location of cropped ‘region of interest’* (ROI) to remove roots touching the column wall; (D) cropped image showing location of root; (E) Annotated image of soil matrix, plant root and pore space; (F) Image highlighting soil matrix surface following calibration using global thresholding operation in VGStudio Max software (Volume Graphics, GmbH, Germany); (G)+(H) Images highlighting surface of root before and after cleaning operation (morphological closing filtering); (I)+(J) show the surface of an iterative dilation process (+2) to create a series of regions of interest moving from the root surface into the ‘rhizosphere soil’. A total of 17 regions were created and analysed for total soil porosity in 3-D. (K)+(L) Magnified images showing the first four dilations from the root surface. The region coloured red in (K) is disregarded from the porosity measurements to allow for errors in segmentations and partial volume effects. (L) Region coloured green highlights the first region used in the analysis. Data for each sequential region from the root surface is presented in Figure 4. Scale bar (A-D) = 6.25 mm; (E-J) = 0.3 mm; (K-J) = 0.1 mm.

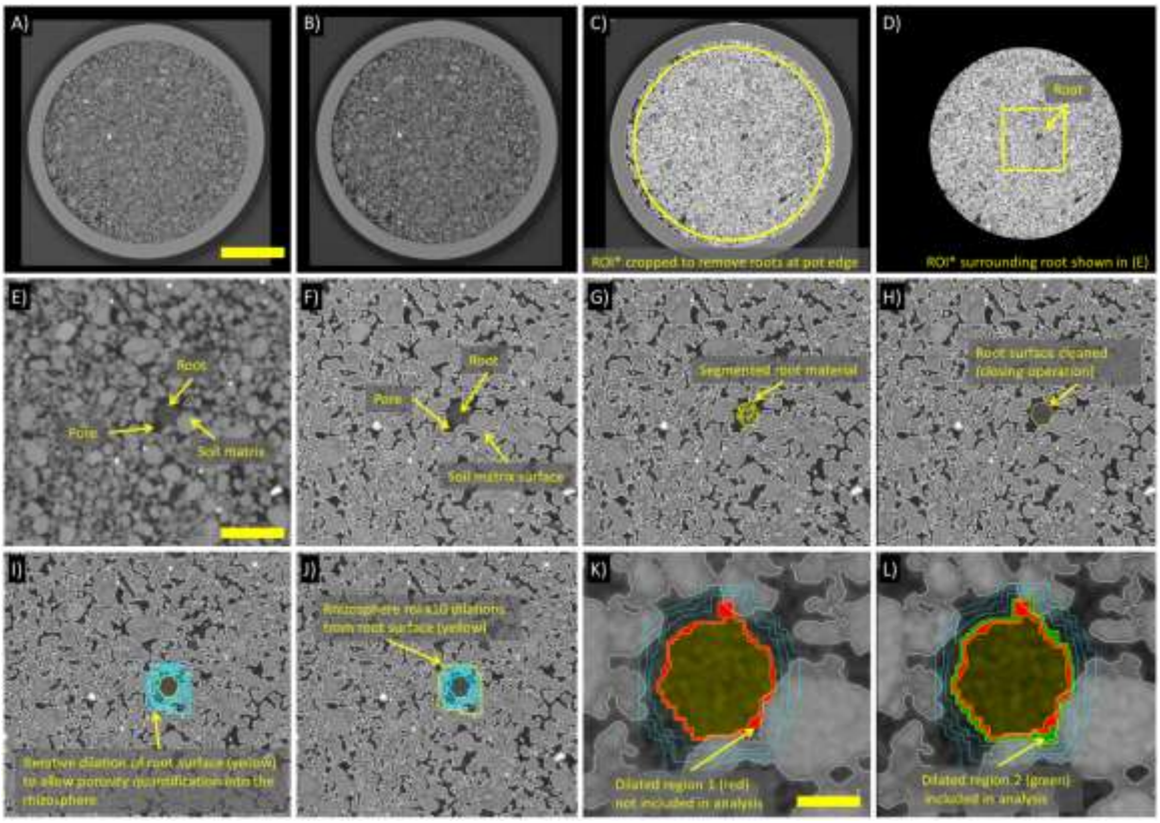

Figure S4 - Example X-ray CT images to demonstrate the change in soil porosity due to the insertion of an artificial root (nylon wire) in a clay loam soil column (70 mm height x 25 mm diameter). Panel A, C (XY slices) and E (ZX slice) show the soil structure before (T0) insertion of the nylon wire. Panel B, D (XY Slice) and E (ZX slice) show the structure after (T1) insertion of the nylon root. The inner and outer zones shown in cyan in panel C and D relate to 2 and 90 voxel region of interest dilations (0.024 and 1mm i.e. rhizosphere and bulk soil respectively). The porosity change data with distance from the root surface (method presented in Fig S1) is shown in Figure S2. Scale bars on XY and ZX images are 2 mm and 3 mm, respectively.

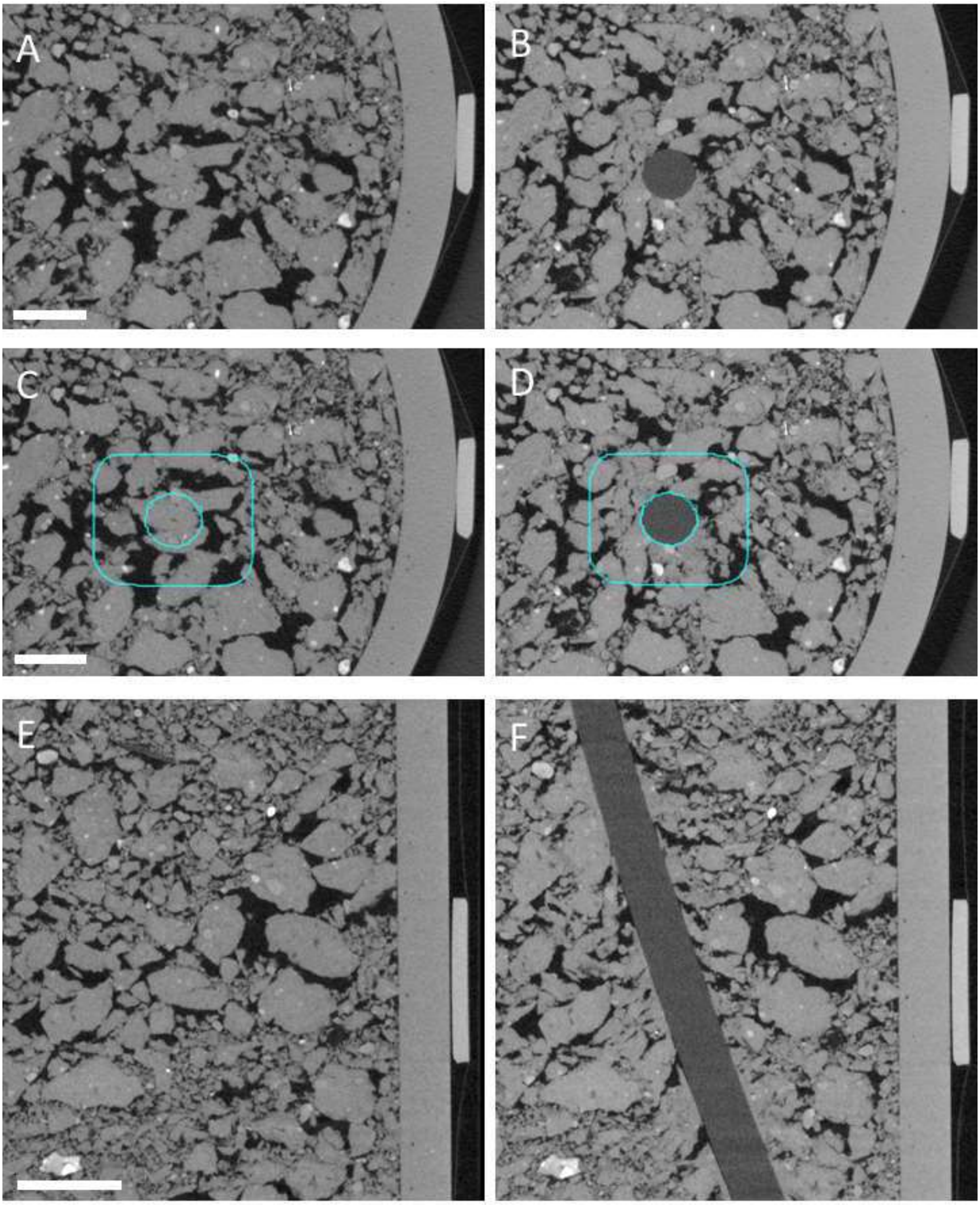

Figure S5 – Examples of supplementary work on control samples to demonstrate the impact of root growth on soil structure dynamics. A) Variation in soil porosity at day 0 (i.e. no root effect) for clay loam and sandy loam soil. Soil porosity was measured as described in Figure S1 but from a hypothetical root surface in the centre of the column at sequential 2 voxel dilations into the bulk soil in VGStudioMAX. B) Variation in porosity from an artificial root (nylon wire) showing the porosity both before (T0) and after (T1) insertion in a packed clay loam soil using the method described in the accompanying paper. Similar trends were observed for the sandy loam soil. Error bars show pooled standard error of the mean.

A

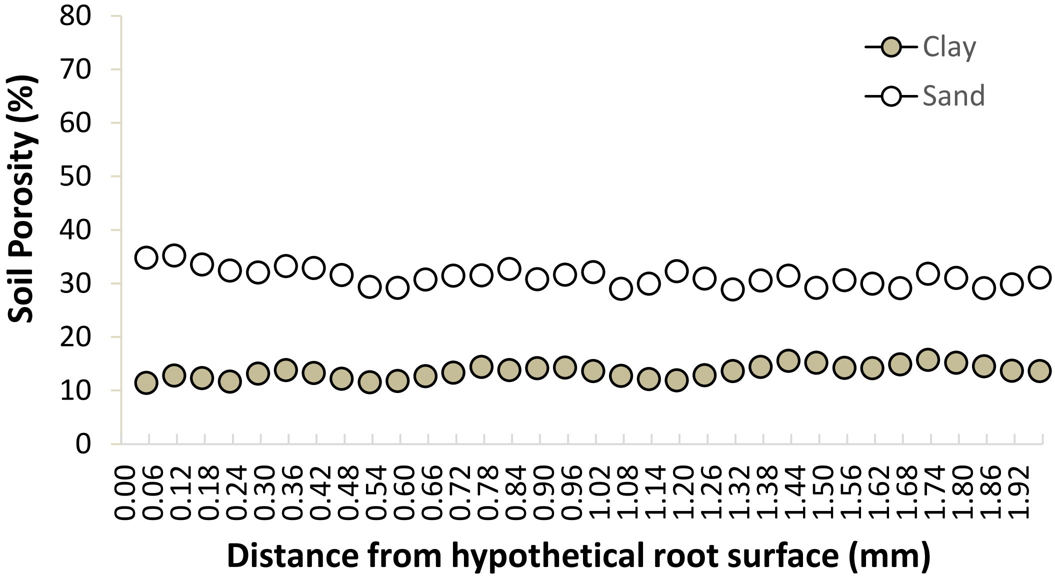

B

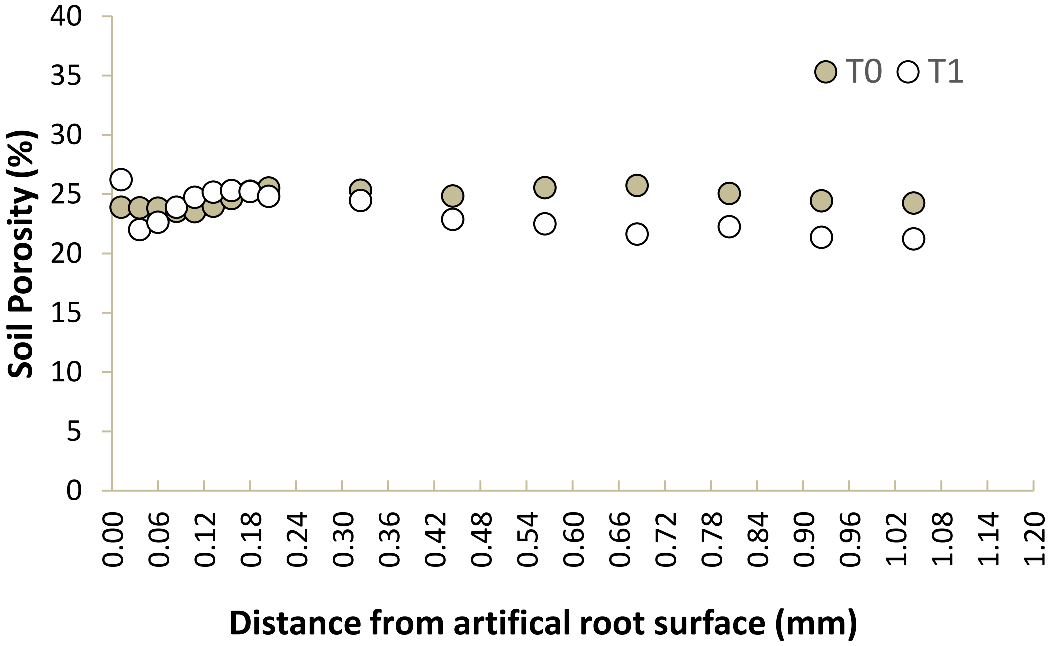

Supplement: Supplementary file 1 — Supplementary Figures [file 41598_2017_14904_MOESM1_ESM.pdf]
